# EHMTI-0031. Efficacy and safety of venlafaxine for the treatment of chronic migraine: a randomized, double-blind, controlled trial

**DOI:** 10.1186/1129-2377-15-S1-G38

**Published:** 2014-09-18

**Authors:** M Togha, F Taghdiri, S Razeghi Jahromi

**Affiliations:** 1 Neurology, Tehran University of Medical Sciences, Tehran, Iran

## Introduction

Chronic migraine (CM) is a disabling neurological disorder that is defined in different ways. Treatment of CM is difficult and needs multidisciplinary approach. To date, minimal study of chronic migraine treatment has been done and few medications are suggested to improve headache duration and intensity in chronic migraine. Different studies demonstrated that topiramate at a dose of 100 mg/day, can reduce migraine days in CM patients when compared with placebo.

## Aims

The purpose of this double-blind, randomized trial was to evaluate and compare the effects of extended-release venlafaxine with topiramate in patients with chronic migraine and medication overuse headache (MOH) to investigate whether venlafaxine could be at least as effective as topiramate.

## Methods

Chronic migraine was defined according to the criteria of the Headache Classification Committee of the International Headache Society (IHS). A prospective, 4-week run-in phase was followed by a 12-week treatment phase which consistent of a 4-week titration and 8-week maintenance period.

## Results

There was no statistically significant difference in terms of primary or secondary efficacy measures between topiramate-group and venlafaxine-group during the double-blind period.

## Conclusions

In this double-blind, randomized study extended-release venlafaxine 150 mg/day found to be effective in chronic migraine. Our study showed a clear effect that was in subgroup of subjects with medication overuse. In addition, in this study, the low number of adverse events showed venlafaxine to be well tolerated. Future controlled trials expended longer with larger sample size, should also support the effectiveness and safety of venlafaxine in patients suffering from chronic migraine.

No conflict of interest.

